# The NLRP3 Inflammasome as a Novel Player of the Intercellular Crosstalk in Metabolic Disorders

**DOI:** 10.1155/2013/678627

**Published:** 2013-06-13

**Authors:** Elisa Benetti, Fausto Chiazza, Nimesh S. A. Patel, Massimo Collino

**Affiliations:** ^1^Department of Drug Science and Technology, University of Turin, Via P. Giuria 9, 10125 Torino, Italy; ^2^Queen Mary University of London, The William Harvey Research Institute, Barts and the London School of Medicine and Dentistry, London EC1M 6BQ, UK

## Abstract

The combination of obesity and type 2 diabetes is a serious health problem, which is projected to afflict 300 million people worldwide by 2020. Both clinical and translational laboratory studies have demonstrated that chronic inflammation is associated with obesity and obesity-related conditions such as insulin resistance. However, the precise etiopathogenetic mechanisms linking obesity to diabetes remain to be elucidated, and the pathways that mediate this phenomenon are not fully characterized. One of the most recently identified signaling pathways, whose activation seems to affect many metabolic disorders, is the “inflammasome,” a multiprotein complex composed of NLRP3 (nucleotide-binding domain and leucine-rich repeat protein 3), ASC (apoptosis-associated speck-like protein containing a CARD), and procaspase-1. NLRP3 inflammasome activation leads to the processing and secretion of the proinflammatory cytokines interleukin- (IL-) 1**β** and IL-18. The goal of this paper is to review new insights on the effects of the NLRP3 inflammasome activation in the complex mechanisms of crosstalk between different organs, for a better understanding of the role of chronic inflammation in metabolic disease pathogenesis. We will provide here a perspective on the current research on NLRP3 inflammasome, which may represent an innovative therapeutic target to reverse the detrimental metabolic consequences of the metabolic inflammation.

## 1. The NLRP3 Inflammasome: An Overview 

**Figure 1:**
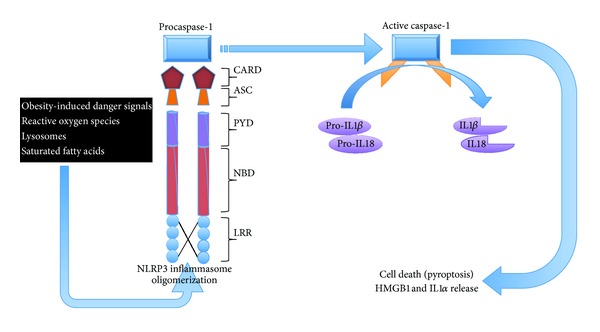
The release of obesity-related danger signals such as reactive oxygen species, lysosomes, and other obesity-induced danger signals resulting in the oligomerization of NLRP3 in adipose tissue. The NLRP3 inflammasome is made up of carboxy terminal leucine-rich repeats (LRRs), a nucleotide-binding domain (NBD), and an N-terminal pyrin domain (PYD). The resulting oligomerization causes the recruitment of procaspase-1 via homotypic binding of caspase activation and recruitment domain (CARD) or through the PYD by means of the adapter apoptosis-associated speck-like protein containing a CARD (ASC). Caspase-1 is therefore activated and initiates the cleavage of prointerleukin (IL)1*β* and pro-IL18 to form the active cytokines IL1*β* and IL18. The activation of caspase-1 also results in pyroptosis (a form of lytic cell death during inflammation) and the release of high mobility group box 1 (HMGB1) and IL1*α*.

The inflammasomes are signaling platforms, which are assembled in response to pathogen-associated and damage-associated molecular pattern molecules and environmental irritants. Currently, inflammasomes are distinguished into two families: the NOD-like receptor (NLR) family and the pyrin and HIN200 (haematopoietic interferon-inducible nuclear antigens with 200 amino-acid repeats) domain-containing protein (PYHIN) family. The NLR family consists of NLRP1, NLRP2, NLRP3, NLRP6, NLRC4, and NLRP12. The PYHIN family consists of AIM2 and IFI16. Each inflammasome is induced by numerous different exogenous and endogenous signals. This review will focus on the NLRP3 inflammasome. The NLRP3 inflammasome is a multiprotein, large cytoplasmic complex (>700 kDa), composed of a specific member of the NOD-like receptor protein (NLRP) subfamily, the adaptor protein named apoptosis-associated speck-like protein containing a CARD (ASC), and procaspase-1, which are preferentially expressed in adipose tissue macrophages (ATMs) [1]. Unlike the typical signaling cascades downstream of many innate receptors such as other NLRP members, the NLRP3 inflammasome is a proteolytic caspase-1-activating platform. The activation of NLRP3 leads to oligomerization and recruitment of ASC. NLRP3 contains an N-terminal pyrin domain (PYD), which is used to physically interact with the PYD domain of ASC, thus facilitating the subsequent recruitment and activation of procaspase-1. Caspase-1 is then autocatalytically cleaved to its active form (Figure 1). Caspase-1 does not play a major role in apoptosis. Instead, once activated, caspase-1, as far as we are currently aware, cleaves the proforms of two potent proinflammatory cytokines interleukin- (IL-) 1*β* and IL-18 in the cytoplasm. This has two main effects; firstly it activates the two cytokines and secondly in this mature form these cytokines can be released from the cell. The active form of caspase-1 also has the ability to induce the release of IL-1*α* and HMGB-1 (high mobility group box 1), as well as initiate a lytic form of cell death called pyroptosis [2–4] (Figure 1). The primary role of the inflammasome and its products seems to be as part of the body's innate immune system, in that they can be triggered to assist in the defense against invading pathogens. Indeed much of the data published on the inflammasome/caspase 1 is on its role in the body's response to microbial molecules (bacterial, fungal, or viral) with conserved molecular structures known as “pathogen associated molecular patterns” (PAMPs) [5, 6]. In addition to PAMPs, the NLRP3 inflammasome is also proficient in sensing stress to endogenous (nonmicrobial) danger signals (“danger associated molecular patterns,” DAMPs) from damaged cells. DAMPs can include molecules such as reactive oxygen species (ROS), adenosine triphosphate (ATP), hypotonic stress, uric acid crystals, or noxious exogenous factors such as environmental insults, asbestos, and UV radiation [7].

There are a number of potential mechanisms for the assembly of the NLRP3 inflammasome, as described earlier. According to one hypothesis, mitochondria are the principal source of reactive oxygen species (ROS) required for inflammasome activation; several recent studies have implicated ROS produced by mitochondria, rather than phagosomes, in NLRP3 activation exerting an indirect effect on pathways of metabolism [8, 9]. A second mechanism involves the disruption of lysosomal membrane integrity by crystalline materials and peptide aggregates [10, 11]. Upon uptake of such substances, lysosomal rupture leads to the leakage of lysosomal proteases, specifically cathepsins B and L, into the cytosol where they could possibly mediate NLRP3 inflammasome activation by an as-yet-undefined cleavage event. In addition, type-2 diabetic patients and mice fed a high-fat diet demonstrate IL-1*β* production following inflammasome activation from obesity-induced danger signals [12]. Mice have also been shown to become glucose intolerant following activation of the inflammasome in hematopoietic cells by the saturated fatty acid palmitate [13]. Very recently, Vajjhala and colleagues [14] have shed light on the molecular details of the complex mechanisms of NLRP3 inflammasome assembly and activation, identifying multiple binding sites on the PYD domain of the adaptor protein ASC which allow self-association and interaction with binding partners.

## 2. The NLRP3 Inflammasome in Obesity and Type 2 Diabetes

Several *in vitro*, *in vivo* studies and clinical trials provide evidence that supports a causative role of IL-1*β* in the pathogenesis of type 2 diabetes [15], and elevation in circulating levels of IL-1*β* predicts type 2 diabetes when combined with serum IL-6 levels [16]. Prolonged IL-1*β* treatment has been demonstrated to reduce the insulin-induced glucose uptake in murine adipocytes [17]. In contrast, addition of the IL-1*β* receptor antagonist to adipocytes resulted in increased insulin sensitivity as reflected by increased levels of phosphorylated AKT in response to insulin. Similarly, IL-1*β* can inhibit the insulin-stimulated glycogen synthesis in rat hepatocytes [18]. These results were confirmed by showing that IL-1*β* knockout mice were more insulin sensitive as compared to wild-type control animals [19]. In humans, elevated plasma levels of IL-1*β* have been found to be predictive of type 2 diabetes [16], and clinical studies have suggested that treatment with the IL-1*β* receptor antagonist anakinra has beneficial effects in type 2 diabetic patients [20]. A number of recent landmark studies have pointed out a key role for an excessive NLRP3 inflammasome activation in the IL-1*β*-related development of type 2 diabetes. The association between the NLRP3 inflammasome and both insulin resistance and obesity has been suggested by animal studies showing that genetic ablation of NLRP3 improved insulin sensitivity and glucose homeostasis [21]. Specifically, adipocytes isolated from NLRP3-deficient mice showed an increase in insulin sensitivity as determined by phosphorylation of Akt. In line with the rise in insulin sensitivity, IL-1*β* production of adipose tissue isolated from NLRP3 knockout mice was significantly reduced as compared to white adipose tissue from wild-type animals. Other studies [12, 13] have shown that improvement in insulin sensitivity (increased phosphorylation of the insulin receptor subtrate-1 and Akt) can also be detected in liver and muscle of NLRP3 knockout mice on a high-fat diet for 12 weeks. This effect was associated with a significant reduction in the tissue mRNA expression of inflammatory cytokines compared to wild-type control [13]. Ablation of the NLRP3 in mice has been also reported to protect from obesity-associated macrophage activation in adipose tissue, reducing M1-like macrophage gene expression (tumor necrosis factor-*α*, chemokine ligand 20, and chemokine ligand 11) and increasing the expression of M2-like cytokines (interleukin-10). This effect was associated with an increase in the number of M2 macrophages in NLRP3-deficient obese mice, without affecting the M1 macrophage frequency [12]. To confirm the clinical relevance of these data generated from mouse models, the same authors have demonstrated that weight loss reduced NLRP3 expression in abdominal subcutaneous adipose tissue in obese patients with type 2 diabetes, which was accompanied by improved glucose homeostasis [12]. Furthermore, strong correlations between the expression of NLRP3 inflammasome-related genes and insulin resistance have been recently reported in obese male subjects with impaired glucose tolerance [22]. Additionally, type 2 diabetic patients showed elevated levels of NLRP3, ASC, IL-1*β*, and IL-18 mRNA and protein expression in monocyte-derived macrophages, compared with those in healthy control subjects. Besides, the cleavage of caspase-1 and release of mature IL-1*β* were significantly elevated in monocyte-derived macrophages from type 2 diabetic patients compared with controls [23]. Inflammatory cytokines are known to contribute crucially to the development of insulin resistance by activating different kinases that disrupt insulin signaling. The endoplasmatic reticulum (ER) is an extensive membrane network which has been recently demonstrated to be involved in the transduction of cytokines effects into activation of different kinases. The early steps of insulin biosynthesis occur in the ER of pancreatic *β* cells, thus further suggesting the key role of ER load and folding activity in the insulin biosynthesis [24]. A major role of the ER is to ensure the synthesis and folding of membrane and secreted proteins, and any disturbance in this function (e.g., excessive protein synthesis or accumulation of unfolded or misfolded proteins in the ER lumen) leads to an “ER stress” response, also known as the unfolded protein response (UPR). The recent literature suggests that ER stress may act directly as a negative modulator of the insulin biosynthesis and insulin signaling pathways but also indirectly by promoting lipid accumulation [25, 26]. ER stress also plays a role in the dysregulation of adipokine secretion by adipose tissue, frequently observed in obesity and insulin resistance [27, 28], and CD14+ monocytes isolated from diabetic patients showed evidence of ER stress, which may underlie the functional defects in these cells [29]. Interestingly, ER stress has been recently demonstrated to activate the NLRP3 inflammasome, resulting in the subsequent release of IL-1*β* by human macrophages, with an activation mechanism similar to that of other known NLRP3 activators, requiring ROS generation and potassium efflux [30]. The thioredoxin-interacting protein (TXNIP), a critical node in the development of ER stress leading to programmed cell death of pancreatic *β* cells, activates the NLRP3 inflammasome, causing procaspase-1 cleavage and IL-1*β* secretion in human monocytic cells [31]. The role of ER stress in promoting NLRP3 inflammasome activation is consistent with the subcellular localization of NLRP3. In resting cells, NLRP3 is associated with ER membranes, and then upon activation NLRP3 is redistribute to the perinuclear space where it colocalizes with endoplasmic reticulum and mitochondria organelle clusters [9]. 

## 3. The NLRP3 Inflammasome and the Organ Crosstalk in the Metabolic Inflammation

NLRP3 inflammasome plays a substantial role in sensing obesity-associated inducers of caspase-1 activation and therefore regulates the magnitude of the inflammation and its downstream effects on insulin signaling in different organs, as reported here later.

### 3.1. Immune Effector Cells

NLRP3 expression is detected mainly in the cytosol of granulocytes, monocytes, dendritic cells, T and B cells, and osteoblasts [32]. Thus, most of the first studies characterizing the role of NLRP3 signaling have been conducted in cells of the immune system. Several studies on innate immune cells have demonstrated that the myeloid-derived NLRP3 inflammasome complex may contribute to promote inflammatory cytokine production and insulin resistance through reduction of insulin signaling. *In vitro* experiments have shown that elevated concentration of saturated fatty acids (SFAs), caused by a high-fat diet, may activate the NLRP3 inflammasome in macrophages through a newly identified AMP-activated protein kinase and unc-51-like kinase-1 autophagy signaling cascade [13]. Besides, both *ex vivo* and *in vivo* exposure of bone marrow derived dendritic cells to dietary SFA resulted in increased NLRP3 inflammasome activation and reduced adipocyte insulin sensitivity. More specifically, dietary SFA may act as a primer of the NLRP3 inflammasome protein complex enhancing NLRP3, caspase-1, and pro-IL-1*β* mRNA expression. A second signal is then required to induce maturation of IL-1*β* from inactive pro-IL-1*β*. This second step can be triggered by exposure to ATP, ROS, or ceramide [33]. Overall, these data suggest that exposure to dietary SFA represents the key metabolic stressor relevant to both priming and processing of IL-1*β* in both adipocytes and innate immune cells. However, it must be stressed that the high expression of NLRP3 in primary adipocyte fractions of enzymatically digested adipose tissue may be attributable in large part to lipid-laden macrophages that contaminate enriched adipocyte fractions, as also suggested by immunofluorescence and qRT-PCR data, showing that NLRP3 is highly expressed in adipose tissue macrophages with low expression in adipocytes [12]. Besides, standard isolation procedures for isolating primary adipose cells often involve collagenase digestion, which have been shown to be a potent inducer of cytokine gene transcription and protein secretion [34]. These findings highlight a new model of organ crosstalk, in which leukocyte and macrophage recruitment in key insulin target tissues, such as liver, adipose, and muscle, may promote insulin resistance by enhancing inflammasome activation. This is in keeping with recent studies showing that IL-1*β*'s role in regulating the endocrine function of adipose tissue is mediated by its own ability to evoke local macrophage recruitment and lipid accumulation in an autocrine/paracrine manner [35]. As in diabetic patients pancreas, adipose tissue, liver, and kidney, with infiltrated macrophages, are major sites of origin of inflammation, it might be intriguing to investigate the specific contribution of NLRP3 inflammasome activation in these different insulin target tissues and to identify the specific inducers that selectivity participate in the mechanism of tissue NLRP3 inflammasome activation. 

### 3.2. Pancreas

Pancreatic islets of type 2 diabetic patients have amyloid deposition and increased production of proinflammatory cytokines and chemokines. The unique, primary component of islet amyloid deposits is the islet amyloid polypeptide (IAPP; also known as amylin). Mice overexpressing IAPP produce higher amounts of IL-1*β* [36], and exposure to high levels of IL-1*β* has been demonstrated to induce beta cell death in cell culture, interfering with signaling to NF-*κ*B through IKK*β* or the I*κ*B*α* super-repressor [37]. In keeping with these results, neutralizing IL-1*β* on isolated beta cells using IL-1 receptor antagonist significantly improves *β*-cell survival [38]. However, the precise mechanism(s) by which IL-1*β* affects pancreatic *β*-cell failure is still debated. Zhou et al. [39] were the first to identify a possible signaling pathway involved in NLRP3 inflammasome activation under conditions of metabolic stress. They showed that thioredoxin-interacting protein (TXNIP), also known as vitamin D3 upregulated protein 1 (VDUP1), is an upstream and highly selective activating ligand for NLRP3, with no effect on the activity of other inflammasomes (e.g., NLRC4 and AIM2). TXNIP-dependent NLRP3 inflammasome activation drives IL-1*β* secretion from pancreatic islets in response to chronic elevated glucose, thus suggesting, for the first time, that NLRP3, activated under conditions of metabolic stress, mediates IL-1*β*-driven islet failure. Other authors have identified oligomers of IAPP, as a key trigger for NLRP3 inflammasome activation and the following processing of IL-1*β* [40]. Obesity-induced pancreatic *β*-cell death is regulated, at least in part, by the NLRP3 inflammasome, as demonstrated in NLRP3-deficient mice in late-stage obesity, where the ablation of NLRP3 is associated with reduced cell death and increase in pancreatic islet size and local insulin levels [41].

### 3.3. Adipose Tissue

As noted by Vandanmagsar et al., the NLRP3 inflammasome is activated in adipose tissue in mouse models of obesity and attenuated by calorie restriction. NLRP3 inflammasome levels also correlate with glycaemia in type 2 diabetes patients after weight loss interventions [12]. Besides, mice deficient in inflammasome components are protected from body-weight gain and adipocyte hypertrophy, induced by chronic exposure to a high-fat diet [1]. NLRP3 inflammasome components have been reported to be abundantly represented in adipocytes of patients with metabolic syndrome, mainly in adipocytes from samples of visceral adipose tissue. In contrast, the inflammasome in subcutaneous adipose tissue adipocytes did not seem to be grossly influenced by the presence of the metabolic syndrome [42]. Interestingly, caspase-1 activation in adipose tissue of obese animals takes place partly independent of macrophage infiltration. Partial depletion of macrophages from adipose tissue of obese animals decreased the expression of the macrophage marker CD68, with no significant alteration in the expression of caspase-1, thus suggesting that the effects of the NLRP3-ASC-caspase-1 protein complex on adipose tissue are not only exerted though infiltrating macrophages. This observation was also confirmed in *in vitro* experiments showing caspase-1 activation in adipocytes in settings free of inflammatory cells [43] and increased insulin sensitivity in adipocytes lacking of caspase-1 or the inflammasome component NLRP3 [21]. In addition, adipocyte upregulation of IL-1*β* expression and secretion in response to inflammatory stimuli has been shown to induce hepatic insulin resistance, thus suggesting a further intriguing role for NLRP3 inflammasome activation in the dysfunctional communication between adipocytes and hepatocytes [35, 43]. Overall these data reveal a novel metabolic function of the NLRP3 inflammasome in adipose tissue, suggesting that its pharmacological modulation in obese and/or patients with type 2 diabetes may restore the metabolic function of adipose tissue and subsequently improve insulin sensitivity.

### 3.4. Liver

The involvement of the inflammasome in nonalcoholic fatty liver disease (NAFLD) and non-alcoholic steatohepatitis (NASH) is slowly being elucidated. The presence of NLRP3 inflammasome and/or inflammasome activation has been shown in sinusoidal endothelial cells [44], stellate cells [45], and hepatocytes [46]. Recently, inflammasome activation has been associated with NASH, and long-term high-fat diet administration resulted in reduced hepatic steatosis in NLRP3 knockout mice [12]. Selective deficiency in IL-1*β* in liver parenchymal cells, but not in bone-marrow-derived cells, protected mice from diet-induced steatohepatitis and fibrosis [47]. Increased mRNA expression of NLRP3 inflammasome components was found in human livers of NASH patients [46] where NLRP3 levels were decreased after weight loss. These observations suggest that inflammasome activation by different cell types may contribute to different aspects of steatohepatitis. In contrast, to date, there are no data suggesting a potential role of NLRP3 inflammasome activation on impaired glycogen synthesis and/or augmented glycogenolysis.

### 3.5. Gut

There is evidence that the inflammasome components are important in the maintenance of the integrity of the intestinal epithelium and the defense against pathogenic organisms that can invade the gastrointestinal tract. For instance, mice lacking the inflammasome components NLRP3 and caspase-1 are hypersusceptible to gastrointestinal inflammation induced by Citrobacter rodentium, an enteric bacterial pathogen of the mouse intestinal tract that triggers inflammatory responses resembling those of humans infected with enteropathogenic and enterohemorrhagic *Escherichia coli*. The increased host susceptibility to *C. rodentium* is due to the failure to produce normal levels of IL-1*β* and IL-18 in the presence of NLRP3 and *Caspase-1* deficiency [48]. NLRP3-deficient mice had been reported to show increased susceptibility to dextran-sulfate-sodium- (DSS-) induced colitis with increased mortality and weight loss in three different studies [49–51]. However, other authors did not show a negative regulatory effect of NLRP3 on colitis, showing that NLRP3-null mice or mice pretreated with the caspase-1 inhibitor pralnacasan had less severe colitis when treated with DSS, which was related to decreased IL-1*β* secretion of DSS-exposed NLRP3-deficient macrophages *in vitro* [52, 53]. This discrepancy could be due not only to differences in protocols but also to baseline differences in the gut microbiota that might account for the dissimilar phenotypes. The crucial role of inflammasome components in the impairments of the gut microbiota composition is also suggested by recent studies demonstrating that NLRP3 inflammasome regulates the gastrointestinal microbiome and can thereby affect host susceptibility to diseases beyond the gastrointestinal tract, including obesity and diabetes. In particular, modulation of the intestinal microbiota through multiple inflammasome components has been recently demonstrated to be a critical determinant of NAFLD/NASH progression as well as multiple other aspects of metabolic syndrome such as weight gain and glucose homeostasis [54]. Inflammasome-deficiency-associated changes in the configuration of the gut microbiota are associated with exacerbated hepatic steatosis and inflammation through influx of TLR4 and TLR9 agonists into the portal circulation, leading to enhanced hepatic tumour-necrosis factor- (TNF-) *α* expression that drives NASH progression [54]. 

### 3.6. Kidney

Little is known of the role of the NLRP3 inflammasome complex in the development of renal metabolic damage. In humans, IL-18 and caspase-1 are expressed in renal tubular epithelium, and patients with chronic kidney disease or the nephrotic syndrome exhibit elevated levels of IL-18 [55–57]. In a cohort of renal biopsies from patients with nondiabetic kidney disease, levels of mRNA encoding NLRP3 correlate with renal function [58], strongly suggesting that NLRP3 contributes to the pathogenesis of chronic kidney disease. This is supported by experimental data showing that inflammasome-regulated cytokines such as IL-1*β* and IL-18 are implicated in animal models of chronic kidney disease, including glomerulonephritis and renal ischemic injury [59]. In an animal study aimed to evaluate the renal consequences of the chronic administration of high-fructose corn syrup (HFCS-55), the major sweetener in foods and soft-drinks, we have recently demonstrated that HFCS-55 feeding caused a significant increase in body weight and more importantly dyslipidemia, hyperinsulinemia, and an increase in insulin resistance due to impaired insulin signaling [60]. Most notably, the HFCS-55 diet evoked upregulation of renal NLRP3 expression, resulting in activation of caspase-1 and the subsequent cleavage of pro-IL1*β* to the biologically active secreted form IL-1*β*. These effects were due, at least in part, to the marked hyperuricemia afforded by the dietary manipulation, as also confirmed by a previous study demonstrating that increased levels of uric acid directly activate the NLRP3 inflammasome [61]. Similarly, rats fed with fructose, which is known to raise uric acid levels, showed a significant increase in renal protein levels of NLRP3 [62]. However, one important question that remains to be answered regards the specific cell types involved in renal NLRP3 activation. As several studies have shown that monocyte/macrophage recruitment to the kidney significantly contributes to the renal injury, we cannot rule out that the increased NLRP3 activation in the kidney is due to an increase in the infiltrating macrophages or, more likely, to a crosstalk between macrophages and tubular/glomerular cells.

### 3.7. Skeletal Muscle

Although it has been recently proposed that sarcopenia (loss of muscle mass) and myosteatosis (fat infiltration in skeletal muscle) exert a key role in triggering insulin resistance in obese patients, so far the potential role of NLRP3 complex activation in muscle activity and muscle production of inflammatory mediators has not yet been investigated. However, there is evidence that components of the inflammasome complex are upregulated in dysferlin-deficient human muscle, thus suggesting that skeletal muscle cells can actively participate in inflammasome activation [63]. This is a crucial point as recent studies have demonstrated that skeletal muscle cells produce and release cytokines (myokines) that act in an autocrine, paracrine, and/or endocrine manner to modulate metabolic and inflammatory process. For example, it has been demonstrated very recently that muscular expression of PGC-1 alpha stimulates the secretion of a newly identified myokine, irisin, which improves glucose homeostasis and causes weight loss [64]. However, the interactions between local NLRP3 expression/activity and myokines production as well as the effects of these interactions on muscle structure, function, and insulin-sensitivity in animals and humans have never been investigated. Interestingly, both IL-1*β* and IL-18 seem to exert a crucial role also in the initiation and progression of the idiopathic inflammatory myopathies, a heterogeneous group of chronic disorders with predominant inflammation in muscle tissue, including dermatomyositis, polymyositis, and myositis [65–67]. Studies elucidating the detailed involvement of muscular inflammasome protein complex may thus provide promising targets for new therapies for this heterogeneous group of inflammatory muscle diseases.

## 4. NLRP3 Activation and Cardiovascular Complications of Metabolic Disorders

Individuals with obesity and insulin resistance have an increased burden of cardiovascular disease (CVD). In the Kuopio Ischemic Heart Disease study, Lakka et al. [68] reported a 4.26-fold relative risk for mortality due to heart disease and a 1.77 relative risk for all-cause mortality in obese, insulin-resistant patients. Similarly, in the Botnia study the risk for coronary heart disease (CHD) and stroke was shown to be increased threefold and the risk for cardiovascular mortality was increased six fold [69]. The Hoorn Study examined 615 men and 749 women aged 50 to 75 years without diabetes or a history of CVD at baseline and reported that the development of insulin resistance and/or obesity was associated with about a twofold increase in age-adjusted risk of fatal CVD in men and nonfatal CVD in women [70]. The pathophysiological mechanism by which metabolic disorders increase cardiovascular risk remains under debate. Studies published during the past decade have convincingly demonstrated a pathophysiological role for the inflammatory response in the development of both insulin resistance and related CVD. The finding a little over a decade ago that the secretion of IL-1*β* and IL-18 was increased in an ischemia/reperfusion (I/R) model of suprafused human atrial myocardium [71] provided the first clear link between inflammasome activation and CVD development. Experimental studies in mice with genetic deletion of caspase-1 have identified caspase-1 inhibition as a potential target for pharmacological intervention in the setting of CVD [72–74]. A recent report described formation of the inflammasome in a mouse model of myocardial I/R, mainly in cardiac fibroblasts and infiltrating cells, and reported that ASC knockout mice were protected, with a significant decline in cardiac infiltration of phagocytes, inflammatory cytokine levels, infarct size, and myocardial fibrosis and dysfunction [75]. The inflammasome was also detected in cardiomyocytes bordering the infarct zone during the infarct process, and prevention of inflammasome activation limited infarct size and cardiac enlargement after acute myocardial I/R injury in the mouse [76]. NLRP3 deficiency protects mice also from renal I/R injury [77, 78]. Both studies showed that the absence of NLRP3 protected kidneys against renal I/R injury to a greater extent than the absence of ASC, suggesting that NLRP3 may play an additional role in renal I/R injury independently of ASC and caspase-1. Overall, the ability of members of the NLRP3 inflammasome protein complex to target molecular and cellular pathways involved in both metabolic and cardiovascular diseases suggest that selective pharmacological modulation of NLRP3 inflammasome has the potential to exert synergistic effects in the control of metabolic disorders and its cardiovascular complications. Thus, this unique therapeutic strategy could decrease the burden of cardiovascular morbidity and mortality in the presence of obesity and insulin resistance, although, to date, there are no clinical data to support this concept.

## 5. Conclusions

In conclusion, NLRP3 inflammasome is a novel protein complex that integrates multiple exogenous and endogenous danger signals into the immediate secretion of IL-1*β* and IL-18. Most recent data suggest that activation of the NLRP3 inflammasome complex contributes to the pathophysiological mechanisms that explain the development of visceral obesity and insulin resistance. Thanks to its wide distribution in different tissues and organs, the NLRP3 inflammasome protein complex may represent a crucial signaling pathway that facilitates organ crosstalk and local injury in tissues target of metabolic damage. A better understanding of this novel pathway could help to clarify the crucial role of the molecular mechanisms of interorgan crosstalk during obesity and insulin resistance development. Studies using animal models and human biopsies will be useful to determine the spatial and temporal expression of inflammasome components inside the organs and to correlate these findings with disease activity or prognosis. Gene polymorphism studies on suitable patient cohorts could help to determine the functional significance of the protein expression data. Finally, the identification of selective pharmacological tools able to affect expression and/or activity of this novel pathway could represent the ultimate proof of significance of the inflammasome-caspase-1-IL-1*β*/18 axis in the development of metabolic inflammation. The effects evoked by these novel pharmacological tools should be compared with effects obtained by targeting selective cytokine receptor activities in order to better elucidate the potential crucial role of NLRP3 inflammasome protein complex in mediating inflammatory diseases. This approach may not only offer a potentially fruitful area of research, but it will also hopefully lead to novel and specific therapies for obesity-related conditions such as insulin resistance and its associated cardiovascular complications. 

## References

[1] Stienstra R, van Diepen JA, Tack CJ (2011). Inflammasome is a central player in the induction of obesity and insulin resistance. *Proceedings of the National Academy of Sciences of the United States of America*.

[2] Ting JP-Y, Willingham SB, Bergstralh DT (2008). NLRs at the intersection of cell death and immunity. *Nature Reviews Immunology*.

[3] Yeretssian G, Labbé K, Saleh M (2008). Molecular regulation of inflammation and cell death. *Cytokine*.

[4] Atianand MK, Rathinam VA, Fitzgerald KA (2013). SnapShot: inflammasomes. *Cell*.

[5] Franchi L, Muñoz-Planillo R, Reimer T, Eigenbrod T, Núñez G (2010). Inflammasomes as microbial sensors. *European Journal of Immunology*.

[6] Kanneganti T-D (2010). Central roles of NLRs and inflammasomes in viral infection. *Nature Reviews Immunology*.

[7] Chen GY, Nuñez G (2010). Sterile inflammation: sensing and reacting to damage. *Nature Reviews Immunology*.

[8] Nakahira K, Haspel JA, Rathinam VAK (2011). Autophagy proteins regulate innate immune responses by inhibiting the release of mitochondrial DNA mediated by the NALP3 inflammasome. *Nature Immunology*.

[9] Zhou R, Yazdi AS, Menu P, Tschopp J (2011). A role for mitochondria in NLRP3 inflammasome activation. *Nature*.

[10] Halle A, Hornung V, Petzold GC (2008). The NALP3 inflammasome is involved in the innate immune response to amyloid-*β*. *Nature Immunology*.

[11] Hornung V, Bauernfeind F, Halle A (2008). Silica crystals and aluminum salts activate the NALP3 inflammasome through phagosomal destabilization. *Nature Immunology*.

[12] Vandanmagsar B, Youm Y-H, Ravussin A (2011). The NLRP3 inflammasome instigates obesity-induced inflammation and insulin resistance. *Nature Medicine*.

[13] Wen H, Gris D, Lei Y (2011). Fatty acid-induced NLRP3-ASC inflammasome activation interferes with insulin signaling. *Nature Immunology*.

[14] Vajjhala PR, Mirams RE, Hill JM (2012). Multiple binding sites on the pyrin domain of ASC protein allow self-association and interaction with NLRP3 protein. *The Journal of Biological Chemistry*.

[15] Dinarello CA, Donath MY, Mandrup-Poulsen T (2010). Role of IL-1*β* in type 2 diabetes. *Current Opinion in Endocrinology, Diabetes and Obesity*.

[16] Spranger J, Kroke A, Möhlig M (2003). Inflammatory cytokines and the risk to develop type 2 diabetes: results of the prospective population-based European Prospective Investigation into Cancer and Nutrition (EPIC)-Potsdam study. *Diabetes*.

[17] Jager J, Grémeaux T, Cormont M, Le Marchand-Brustel Y, Tanti J-F (2007). Interleukin-1*β*-induced insulin resistance in adipocytes through down-regulation of insulin receptor substrate-1 expression. *Endocrinology*.

[18] Kanemaki T, Kitade H, Kaibori M (1998). Interleukin 1*β* and interleukin 6, but not tumor necrosis factor *α*, inhibit insulin-stimulated glycogen synthesis in rat hepatocytes. *Hepatology*.

[19] Cleary PA, Orchard TJ, Genuth S (2006). The effect of intensive glycemic treatment on coronary artery calcification in type 1 diabetic participants of the diabetes control and complications trial/epidemiology of diabetes interventions and complications (DCCT/EDIC) study. *Diabetes*.

[20] Larsen CM, Faulenbach M, Vaag A (2007). Interleukin-1-receptor antagonist in type 2 diabetes mellitus. *The New England Journal of Medicine*.

[21] Stienstra R, Joosten LAB, Koenen T (2010). The inflammasome-mediated caspase-1 activation controls adipocyte differentiation and insulin sensitivity. *Cell Metabolism*.

[22] Goossens GH, Blaak EE, Theunissen R (2012). Expression of NLRP3 inflammasome and T cell population markers in adipose tissue are associated with insulin resistance and impaired glucose metabolism in humans. *Molecular Immunology*.

[23] Lee HM, Kim JJ, Kim HJ, Shong M, Ku BJ, Jo EK (2013). Upregulated NLRP3 inflammasome activation in patients with type 2 diabetes. *Diabetes*.

[24] Harding HP, Ron D (2002). Endoplasmic reticulum stress and the development of diabetes: a review. *Diabetes*.

[25] Cnop M, Foufelle F, Velloso LA (2012). Endoplasmic reticulum stress, obesity and diabetes. *Trends in Molecular Medicine*.

[26] Flamment M, Hajduch E, Ferre P, Foufelle F (2012). New insights into ER stress-induced insulin resistance. *Trends in Endocrinology & Metabolism*.

[27] Xu L, Spinas GA, Niessen M (2010). ER stress in adipocytes inhibits insulin signaling, represses lipolysis, and alters the secretion of adipokines without inhibiting glucose transport. *Hormone and Metabolic Research*.

[28] Deng J, Liu S, Zou L, Xu C, Geng B, Xu G (2012). Lipolysis response to endoplasmic reticulum stress in adipose cells. *The Journal of Biological Chemistry*.

[29] Komura T, Sakai Y, Honda M, Takamura T, Matsushima K, Kaneko S (2010). CD14+ monocytes are vulnerable and functionally impaired under endoplasmic reticulum stress in patients with type 2 diabetes. *Diabetes*.

[30] Menu P, Mayor A, Zhou R (2012). ER stress activates the NLRP3 inflammasome via an UPR-independent pathway. *Cell Death and Disease*.

[31] Lerner AG, Upton JP, Praveen PV (2012). IRE1alpha induces thioredoxin-interacting protein to activate the NLRP3 inflammasome and promote programmed cell death under irremediable ER stress. *Cell Metabolism*.

[32] Kummer JA, Broekhuizen R, Everett H (2007). Inflammasome components NALP 1 and 3 show distinct but separate expression profiles in human tissues suggesting a site-specific role in the inflammatory response. *Journal of Histochemistry and Cytochemistry*.

[33] Reynolds CM, McGillicuddy FC, Harford KA, Finucane OM, Mills KH, Roche HM (2012). Dietary saturated fatty acids prime the NLRP3 inflammasome via TLR4 in dendritic cells-implications for diet-induced insulin resistance. *Molecular Nutrition & Food Research*.

[34] Ruan H, Zarnowski MJ, Cushman SW, Lodish HF (2003). Standard isolation of primary adipose cells from mouse epididymal fat pads induces inflammatory mediators and down-regulates adipocyte genes. *The Journal of Biological Chemistry*.

[35] Nov O, Shapiro H, Ovadia H (2013). Interleukin-1beta regulates fat-liver crosstalk in obesity by auto-paracrine modulation of adipose tissue inflammation and expandability. *PLoS One*.

[36] Mandrup-Poulsen T (2010). IAPP boosts islet macrophage IL-1 in type 2 diabetes. *Nature Immunology*.

[37] Collier JJ, Burke SJ, Eisenhauer ME (2011). Pancreatic *β*-cell death in response to pro-inflammatory cytokines is distinct from genuine apoptosis. *PLoS ONE*.

[38] Ardestani A, Sauter NS, Paroni F (2011). Neutralizing interleukin-1*β*(IL-1*β*) induces *β*-cell survival by maintaining PDX1 protein nuclear localization. *The Journal of Biological Chemistry*.

[39] Zhou R, Tardivel A, Thorens B, Choi I, Tschopp J (2010). Thioredoxin-interacting protein links oxidative stress to inflammasome activation. *Nature Immunology*.

[40] Masters SL, Dunne A, Subramanian SL (2010). Activation of the NLRP3 inflammasome by islet amyloid polypeptide provides a mechanism for enhanced IL-1*β* 2 in type 2 diabetes. *Nature Immunology*.

[41] Youm Y-H, Adijiang A, Vandanmagsar B, Burk D, Ravussin A, Dixit VD (2011). Elimination of the NLRP3-ASC inflammasome protects against chronic obesity-induced pancreatic damage. *Endocrinology*.

[42] Madec S, Rossi C, Chiarugi M (2011). Adipocyte P2X7 receptors expression: a role in modulating inflammatory response in subjects with metabolic syndrome?. *Atherosclerosis*.

[43] Nov O, Kohl A, Lewis EC (2010). Interleukin-1*β* may mediate insulin resistance in liver-derived cells in response to adipocyte inflammation. *Endocrinology*.

[44] Imaeda AB, Watanabe A, Sohail MA (2009). Acetaminophen-induced hepatotoxicity in mice is dependent on Tlr9 and the Nalp3 inflammasome. *The Journal of Clinical Investigation*.

[45] Watanabe A, Sohail MA, Gomes DA (2009). Inflammasome-mediated regulation of hepatic stellate cells. *American Journal of Physiology*.

[46] Csak T, Ganz M, Pespisa J, Kodys K, Dolganiuc A, Szabo G (2011). Fatty acid and endotoxin activate inflammasomes in mouse hepatocytes that release danger signals to stimulate immune cells. *Hepatology*.

[47] Kamari Y, Shaish A, Vax E (2011). Lack of interleukin-1*α* or interleukin-1*β* inhibits transformation of steatosis to steatohepatitis and liver fibrosis in hypercholesterolemic mice. *Journal of Hepatology*.

[48] Liu Z, Zaki MH, Vogel P (2012). Role of inflammasomes in host defense against Citrobacter rodentium infection. *The Journal of Biological Chemistry*.

[49] Allen IC, Tekippe EM, Woodford R-MT (2010). The NLRP3 inflammasome functions as a negative regulator of tumorigenesis during colitis-associated cancer. *Journal of Experimental Medicine*.

[50] Zaki MH, Boyd KL, Vogel P, Kastan MB, Lamkanfi M, Kanneganti T-D (2010). The NLRP3 inflammasome protects against loss of epithelial integrity and mortality during experimental colitis. *Immunity*.

[51] Hirota SA, Ng J, Lueng A (2011). NLRP3 inflammasome plays a key role in the regulation of intestinal homeostasis. *Inflammatory Bowel Diseases*.

[52] Bauer C, Loher F, Dauer M (2007). The ICE inhibitor pralnacasan prevents DSS-induced colitis in C57BL/6 mice and suppresses IP-10 mRNA but not TNF-*α* mRNA expression. *Digestive Diseases and Sciences*.

[53] Bauer C, Duewell P, Mayer C (2010). Colitis induced in mice with dextran sulfate sodium (DSS) is mediated by the NLRP3 inflammasome. *Gut*.

[54] Henao-Mejia J, Elinav E, Jin C (2012). Inflammasome-mediated dysbiosis regulates progression of NAFLD and obesity. *Nature*.

[55] Matsumoto K, Kanmatsuse K (2001). Elevated interleukin-18 levels in the urine of nephrotic patients. *Nephron*.

[56] Lonnemann G, Novick D, Rubinstein M, Dinarello CA (2003). Interleukin-18, interleukin-18 binding protein and impaired production of interferon-*γ* in chronic renal failure. *Clinical Nephrology*.

[57] Gauer S, Sichler O, Obermüller N (2007). IL-18 is expressed in the intercalated cell of human kidney. *Kidney International*.

[58] Vilaysane A, Chun J, Seamone ME (2010). The NLRP3 inflammasome promotes renal inflammation and contributes to CKD. *Journal of the American Society of Nephrology*.

[59] Anders H-J, Muruve DA (2011). The inflammasomes in kidney disease. *Journal of the American Society of Nephrology*.

[60] Collino M, Benetti E, Rogazzo M (2013). Reversal of the deleterious effects of chronic dietary HFCS-55 intake by PPAR-delta agonism correlates with impaired NLRP3 inflammasome activation. *Biochemical Pharmacology*.

[61] Jin C, Flavell RA (2010). Molecular mechanism of NLRP3 inflammasome activation. *Journal of Clinical Immunology*.

[62] Hu Q-H, Zhang X, Pan Y, Li Y-C, Kong L-D (2012). Allopurinol, quercetin and rutin ameliorate renal NLRP3 inflammasome activation and lipid accumulation in fructose-fed rats. *Biochemical Pharmacology*.

[63] Rawat R, Cohen TV, Ampong B (2010). Inflammasome up-regulation and activation in dysferlin-deficient skeletal muscle. *American Journal of Pathology*.

[64] Boström P, Wu J, Jedrychowski MP (2012). A PGC1-*α*-dependent myokine that drives brown-fat-like development of white fat and thermogenesis. *Nature*.

[65] Lundberg I, Kratz AK, Alexanderson H, Patarroyo M (2000). Decreased expression of interleukin-1alpha, interleukin-1beta, and cell adhesion molecules in muscle tissue following corticosteroid treatment in patients with polymyositis and dermatomyositis. *Arthritis & Rheumatism*.

[66] Tucci M, Quatraro C, Dammacco F, Silvestris F (2006). Interleukin-18 overexpression as a hallmark of the activity of autoimmune inflammatory myopathies. *Clinical and Experimental Immunology*.

[67] Schmidt J, Barthel K, Wrede A, Salajegheh M, Bähr M, Dalakas MC (2008). Interrelation of inflammation and APP in sIBM: IL-1*β* induces accumulation of *β*-amyloid in skeletal muscle. *Brain*.

[68] Lakka H-M, Laaksonen DE, Lakka TA (2002). The metabolic syndrome and total and cardiovascular disease mortality in middle-aged men. *The Journal of the American Medical Association*.

[69] Isomaa B, Almgren P, Tuomi T (2001). Cardiovascular morbidity and mortality associated with the metabolic syndrome. *Diabetes Care*.

[70] van der Heijden AAWA, Ortegon MM, Niessen LW, Nijpels G, Dekker JM (2009). Prediction of coronary heart disease risk in a general, pre-diabetic, and diabetic population during 10 years of follow-up: accuracy of the Framingham, SCORE, and UKPDS risk functions—The Hoorn Study. *Diabetes Care*.

[71] Pomerantz BJ, Reznikov LL, Harken AH, Dinarello CA (2001). Inhibition of caspase 1 reduces human myocardial ischemic dysfunction via inhibition of IL-18 and IL-1*β*. *Proceedings of the National Academy of Sciences of the United States of America*.

[72] Frantz S, Ducharme A, Sawyer D (2003). Targeted deletion of caspase-1 reduces early mortality and left ventricular dilatation following myocardial infarction. *Journal of Molecular and Cellular Cardiology*.

[73] Syed FM, Hahn HS, Odley A (2005). Proapoptotic effects of caspase-1/interleukin-converting enzyme dominate in myocardial ischemia. *Circulation Research*.

[74] Merkle S, Frantz S, Schön MP (2007). A role for caspase-1 in heart failure. *Circulation Research*.

[75] Kawaguchi M, Takahashi M, Hata T (2011). Inflammasome activation of cardiac fibroblasts is essential for myocardial ischemia/reperfusion injury. *Circulation*.

[76] Mezzaroma E, Toldo S, Farkas D (2011). The inflammasome promotes adverse cardiac remodeling following acute myocardial infarction in the mouse. *Proceedings of the National Academy of Sciences of the United States of America*.

[77] Iyer SS, Pulskens WP, Sadler JJ (2009). Necrotic cells trigger a sterile inflammatory response through the Nlrp3 inflammasome. *Proceedings of the National Academy of Sciences of the United States of America*.

[78] Shigeoka AA, Mueller JL, Kambo A (2010). An inflammasome-independent role for epithelial-expressed Nlrp3 in renal ischemia-reperfusion injury. *The Journal of Immunology*.

